# Effects of Acute Systemic Hypoxia and Hypercapnia on Brain Damage in a Rat Model of Hypoxia-Ischemia

**DOI:** 10.1371/journal.pone.0167359

**Published:** 2016-12-01

**Authors:** Wanchao Yang, Xuezhong Zhang, Nan Wang, Jing Tan, Xianhai Fang, Qi Wang, Tao Tao, Wenzhi Li

**Affiliations:** Department of Anesthesiology, Second Affiliated Hospital of Harbin Medical University P. R. China; and Anesthesiology Key Laboratory, Education Department, Harbin Medical University, Heilongjiang Province, P. R. China; Ehime University Graduate School of Medicine, JAPAN

## Abstract

Therapeutic hypercapnia has the potential for neuroprotection after global cerebral ischemia. Here we further investigated the effects of different degrees of acute systemic hypoxia in combination with hypercapnia on brain damage in a rat model of hypoxia and ischemia. Adult wistar rats underwent unilateral common carotid artery (CCA) ligation for 60 min followed by ventilation with normoxic or systemic hypoxic gas containing 11%O_2_,13%O_2_,15%O_2_ and 18%O_2_ (targeted to PaO_2_ 30–39 mmHg, 40–49 mmHg, 50–59 mmHg, and 60–69 mmHg, respectively) or systemic hypoxic gas containing 8% carbon dioxide (targeted to PaCO_2_ 60–80 mmHg) for 180 min. The mean artery pressure (MAP), blood gas, and cerebral blood flow (CBF) were evaluated. The cortical vascular permeability and brain edema were examined. The ipsilateral cortex damage and the percentage of hippocampal apoptotic neurons were evaluated by Nissl staining and terminal deoxynucleotidyl transferase-mediated 2′-deoxyuridine 5′-triphosphate-biotin nick end labeling (TUNEL) assay as well as flow cytometry, respectively. Immunofluorescence and western blotting were performed to determine aquaporin-4 (AQP4) expression. In rats treated with severe hypoxia (PaO_2_ < 50 mmHg), hypercapnia augmented the decline of MAP with cortical CBF and damaged blood–brain barrier permeability (p < 0.05). In contrast, in rats treated with mild to moderate hypoxia (PaO_2_ > 50 mmHg), hypercapnia protected against these pathophysiological changes. Moreover, hypercapnia treatment significantly reduced brain damage in the ischemic ipsilateral cortex and decreased the percentage of apoptotic neurons in the hippocampus after the CCA ligated rats were exposed to mild or moderate hypoxemia (PaO_2_ > 50 mmHg); especially under mild hypoxemia (PaO_2_ > 60 mmHg), hypercapnia significantly attenuated the expression of AQP4 protein with brain edema (p < 0.05). Hypercapnia exerts beneficial effects under mild to moderate hypoxemia and augments detrimental effects under severe hypoxemia on brain damage in a rat model of hypoxia-ischemia.

## Introduction

Hypoxemia (arterial blood O_2_ tension [PaO_2_] < 60 mmHg) is present in approximately 20% of brain injury patients and commonly associated with poor neurological outcomes [1]. The hypoxic factor is one of the most important mechanisms in the development of many pathological processes (strokes, ischemia, cerebral edema/swelling, *etc*.). Therefore, study of the mechanisms of resistance to different types of hypoxia remains an important problem. Several lines of evidence have shown that hypoxic-ischemic (HI) insults trigger a cascade of biochemical, cellular, and pathological events that result in cell injury and death in the brain[2, 3]. Hypoxia and ischemia can disrupt the integrity of the blood–brain barrier (BBB) [4] and thus increase cerebrovascular permeability with a concomitant increase in vasogenic cerebral edema[5, 6]. Growing evidence has demonstrated that neuronal apoptosis, inflammation and BBB damage may account for the higher susceptibility of the developing brain to HI injury [7–10].

Therapeutic hypercapnia is induced by adding carbon dioxide (CO_2_) to inspired gas and considered a new treatment strategy for various lung injury models[11, 12]. Furthermore, hypercapnia has also proven effective for central nervous system ischemia injuries. However, there are conflicting results concerning the protective effect of permissive hypercapnia in HI brain injury in immature animal models [13]. It has been reported that mild hypercapnia (PaCO_2_ of 50–70 mmHg) protects the immature brain from HI insults compared with normocapnia, whereas severe hypercapnia (PaCO_2_ > 100 mmHg) is deleterious [13]. Similarly, we previously demonstrated that mild and moderate hypercapnia (PaCO_2_ of 60–100 mmHg) is neuroprotective after cerebral ischemia, but the neuroprotective effect is not observed with severe hypercapnia (PaCO_2_ > 100 mmHg) [14]. Furthermore, we found that hypercapnia (PaCO_2_ of 80–100 mmHg) improves neurological outcomes via an anti-apoptotic mechanism in adult rats with focal cerebral ischemic injury[15]. Although the therapeutic windows of hypercapnia have been studied in various animal models of brain injury, it remains unclear how hypoxia combined with reduced cerebral blood flow (ischemia) affects the brain at the consistent level of hypercapnia (PaCO2 of 60–80 mmHg).

A clinically relevant hypoxia-ischemia animal model was successfully accomplished using a modification of the Levine preparation[16]. This model of hemispheric global ischemia has been well accepted as a model of stroke in both adult and newborn animals [17–20]. Here we used this rat model of hypoxia-ischemia to test the effect of different degrees of hypoxia and hypercapnia on brain damage.

## Methods and Materials

### Ethics approval

The experimental protocols were approved by the Institutional Animal Care Committee of Harbin Medical University, and all procedures were conducted in strict accordance with the Harbin Medical University guidelines for the care and use of laboratory animals as well as the ARRIVE (Animal Research: Reporting In Vivo Experiments) guidelines for animal research.

### Animals

Adult male Wistar 10-12-week-old rats (weighing 250–300 g) were provided by the Laboratory Animal Center of Harbin Medical University. Animals were housed with standard chow and water *ad libitum*. The rats (n = 180) were randomly assigned to nine groups (n = 20 rats each): sham (S) group, exposed to the air; hypoxia-ischemia (HI) 30, 40, 50, and 60 groups; and the HI30+ hypercapnia, HI40+ hypercapnia, HI50+ hypercapnia, and HI60+ hypercapnia groups. The HI30, 40, 50, and 60 groups were exposed to PaO_2_ 30–39 mmHg, 40–49 mmHg, 50–59 mmHg, and 60–69 mmHg, which was maintained at O_2_ concentrations of 11, 13, 15, or 18% of the gas mixture, respectively. In the HI+ hypercapnia groups, a humidified gas mixture of 8% CO_2_ containing the above O_2_ concentrations was used to maintain PaCO_2_ at 60–80 mmHg.

### Rat model of hypoxia-ischemia

All rats were fasted overnight before the experiments but allowed free access to water. Each rat was anesthetized with an intraperitoneal (i.p.) injection of 30 mg/kg pentobarbital sodium (Abbott, North Chicago, IL, USA). Under anesthesia, the midline of the neck was incised in the longitudinal plane to expose the left common carotid artery (CCA). The artery then was permanently ligated with 4–0 surgical silk. One hour after the left CCA was ligated, mechanical ventilation (Harvard ventilator 683, Natick, MA, USA) was performed using a gas tank containing either room air (sham group) or a gas mixture containing alone O_2_ (HI groups), or O_2_ and CO_2_ (HI+ hypercapnia groups), and N_2_ via tracheostomy for 3 h. The ventilation conditions were: tidal volume, 9 mL/kg; respiratory rate, 45 breaths/min; and inspiratory to expiratory ratio, 1:1. The concentrations of CO_2_ and O_2_ in the respiratory circuit were monitored continuously using a gas monitor (MindaryBeneView T8; Mindray Medical International Limited, ShenZhen, China).

To monitor regional cortical blood flow (rCBF), the skull was fixed in a stereotaxic frame (model 51600; Stoelting Co., Wood Dale, IL, USA), and a cranial window was created over the left parietal hemisphere as previously described [21]. The rCBF was monitored every 30 min by a laser Doppler flowmeter (PeriFluxsystem5000; Preimed AB, BeiJing, China). The proximal part of the left ligated CCA was cannulated with a 24-gauge Teflon cannula (Becton Dickinson, Sparks, MD, USA) to monitor mean arterial pressure (MAP) and collect arterial blood samples. MAP was measured using an MP150 Workstation and analyzed using AcqKnowledge software (BIOPAC Systems Inc., Santa Barbara, CA, USA) according to the manufacturer’s specifications. Arterial blood gases were examined using a Chiron Diagnostics model 248 blood gases/pH analyzer (Bayer Diagnostics, Norwood, MA, USA). The left femoral vein was cannulated with a 22-gauge Teflon cannula to enable a continuous infusion of 0.9% saline for maintenance fluid. All surgical incisions were infiltrated with 0.25% bupivacaine. A heating pad and lamp were used to maintain the rectal temperature at 36.5 ± 0.5°C.

**Fig 1:**
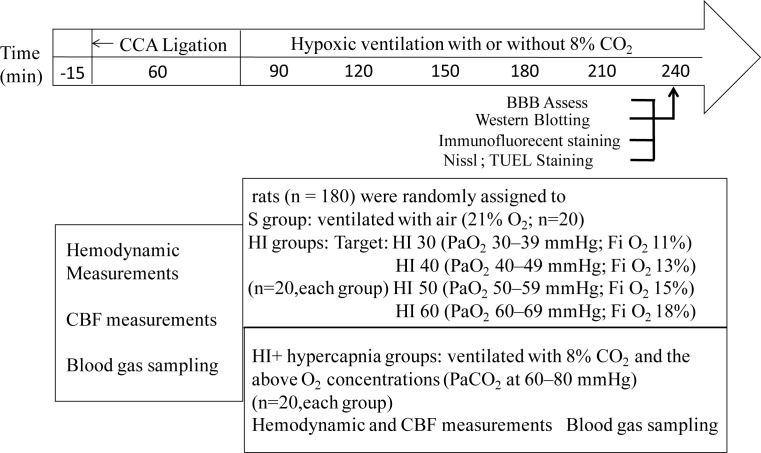
Flow chart. Schematic diagram illustrating the flowchart of the experimental protocol. After the left common carotid artery (CCA) was ligating for 60 min, the rats in the HI30, 40, 50, and 60 groups were exposed to PaO_2_ 30–39 mmHg, 40–49 mmHg, 50–59 mmHg, and 60–69 mmHg, which was maintained at O_2_ concentrations of 11, 13, 15, or 18% of the gas mixture, respectively. In the HI+ hypercapnia groups, a humidified gas mixture of 8% CO_2_ containing the above O_2_ concentrations was used to maintain PaCO_2_ at 60–80 mmHg.

Rats were sacrificed under deeply anesthetized with intravenous injection of 30 mg/kg body weight pentobarbital sodium at 3h after systemic hypoxia with or without hypercapnic ventilation. The brains were quickly removed and used for the following experiments (The experimental protocol is shown in Fig 1).

### Evaluation of BBB permeability

Five rats from each group were randomly selected to assess the BBB permeability. The quantitative evaluation of BBB disruption during HI and hypercapnia was achieved by measuring fluorescence in defined brain areas as previously described[22]. Ten minutes before the end of ventilation with hypoxic and hypercapnic gas, the rats were injected (0.5 mL) with fluorescent dextran (10 kDa in saline 2 mg/mL) via a unilateral cannula implanted into the left common carotid artery. The rats were then transcardially perfused with 0.9% saline to remove the intravascular dextran. The brain was rapidly removed and the left and right cortex was dissected. Tissue was homogenized in 50% wt/vol trichloroacetic acid (Sigma, St. Louis, MO, USA). After centrifugation (10,000 × *g*), the supernatant was collected. The fluorescence intensity (ng/mL) was measured on a microplate fluorescence reader (TECAN Infinite M200, Mӓnnedorf, Switzerland) in 96-well plates and analyzed at an excitation wavelength of 495 nm and an emission wavelength of 520 nm. The differences in the fluorescence intensity between the S and HI groups were calculated as tracer leakage, and the data are presented as percent change from S (normoxic) tissue.

### Measurement of brain water content

After 3 h of hypoxic with or without hypercapnic treatment, five rats from each group were randomly selected. The cerebral cortex of the brain tissue was dissected and placed in a dry culture plate. The cortex was weighted to determine the wet weight and then dried in the oven at 80°C for 48 h to obtain the dry weight. The wet and dry weights were measured using an electronic balance. Brain water content was calculated by the following formula: (wet weight–dry weight)/wet weight × 100%.

### Immunofluorescent staining

After 3 h of hypoxic with or without hypercapnic treatment, three rats from each group were randomly selected. Rats were transcardially perfused with ice-cold phosphate buffered saline (PBS) (pH 7.2) under anesthesia. The brains were removed and sliced into coronal sections (4 μm thick). Brain section was incubated in primary antibodies against AQP-4 (mouse monoclonal antibodies, 1:100 dilution, sc20812; Santa Cruz Biotechnology, Santa Cruz, CA, USA) overnight at 4°C. Sections were then incubated with goat anti-mouse fluorescein-conjugated secondary antibodies (1:1000 dilution, 1031–01; Southern Biotech, Birmingham, USA) for 20 min at 37°C. Images were captured under a laser scanning confocal microscope (Fluoview1000; Olympus, Tokyo, Japan).

### Western blotting analysis

Three samples were obtained from the cerebral cortices in the ischemic ipsilateral to the injury in each group after 3 h of normoxic, hypoxic with or without hypercapnic treatment. Tissues were homogenized in an ice-cold radioimmunoprecipitation buffer with 1 μg/mL of a protease inhibitor cocktail (Thermo Scientific, Rockford, IL, USA). The homogenates were centrifuged at 14,000 rpm for 15 min at 4°C. Protein concentrations in the supernatants were determined by the Bradford protein assay. Proteins were separated by electrophoresis in 8% sodium dodecyl sulfate–polyacrylamide gel and transferred to a nitrocellulose membrane. Membranes were blocked in PBS containing 5% nonfat dry milk and 0.01% Tween-20. The membranes were then incubated with rabbit monoclonal antibody against rat aquaporin-4 (AQP4; 1:200, sc-20812; Santa Cruz Biotechnology) overnight at 4°C. Followed by incubation with goat anti-rabbit horseradish peroxidase–conjugated secondary antibodies (sc-2030; Santa Cruz Biotechnology, Santa Cruz, CA, USA). Glyceraldehyde 3-phosphate dehydrogenase (0.4 mg/mL; Santa Cruz Biotechnology) was used as a loading control. The bands were densitometrically quantified with Image J software (v1.33; National Institutes of Health, Bethesda, MD, USA).

### Histological examination

After 3 h of hypoxic with or without hypercapnia treatment, rats under anesthesia were transcardially perfused with 200 mL of 4% paraformaldehyde in 0.1 M PBS (pH 7.4). The rat brains were removed, post-fixed in 4% paraformaldehyde overnight, and paraffin-embedded, and cut into 4-μm coronal sections for Nissl or terminal deoxynucleotidyl transferase-mediated dUTP-nick end labeling (TUNEL) staining. Apoptosis in the CA1 regions of the hippocampus was assessed using a TUNEL assay performed with an In Situ Apoptosis Detection Kit (Roche Diagnostics, Indianapolis, IN, USA) according to a previously described methodology[23]. As directed by the product specifications, the sections were deparaffinized and rehydrated by heating at 60°C for 10 min. These sections were then incubated in proteinase K (20 μg/ml) for 15 min, followed by 10min quenching in 3% hydrogen peroxide at room temperature. After three 10 min washes in PBS, the sections were incubated with TUNEL reaction mixture for 1 h at 37°C. Sections were washed in PBS three times for 10 min each and then color development was performed in the dark with DAB, which was used as a nuclear counter stain (Beijing Zhongshan Golden Bridge Biotechnology Co. Ltd., Beijing, China). The number of TUNEL positive CA1 neurons was counted carefully in five sections per animal. Cell counts from the hippocampus on each of the five sections were averaged to provide the mean value.

### Flow cytometry Analysis

Apoptosis in the whole hippocampal area was assessed by an Annexin V-FITC/propidium iodide (PI) dual staining kit (556547, FITC Annexin V Apoptosis Detection Kit I, BD Biosciences, USA) according to the manufacturer’s instructions. Analyses were performed with Cell Quest Pro software (BD Biosciences, San Jose, CA) and flow cytometry (BD FACSCalibur^TM^ Flow Cytometry, BD Biosciences, USA.). For each sample, data from10,000 cells were recorded in list mode on logarithmic scales. The upper-right and lower-right quadrants represented late apoptotic neurons, and early apoptotic neurons, respectively. The percentage of apoptotic cell was calculated using 100 × (early + late) apoptotic neurons number/total neurons number.

### Statistical analysis

The statistical analysis was performed using the SPSS software (version 13.01S; Chicago, IL, USA). Data are expressed as mean ± standard deviation. One-way analysis of variance (ANOVA) was used to evaluate the differences in variables among the groups followed by a post-hoc test. Repeated-measures ANOVA were used to determine the time-dependent difference within the same group followed by a post-hoc test. All p values are two-tailed and those <0.05 were considered significant.

## Results

### Physiological data

Table 1 summarizes the MAP and pH at baseline, 60 min after the CCA ligation, and 60, 120, and 180 min after ventilation during hypoxic with or without hypercapnic mixture gas in the rats from each group. There were no deaths related to the H/I procedure. At baseline and 60 min after the CCA ligation, there were no significant differences in MAP and pH among the nine groups (p > 0.05). Compared with the baseline values, the MAP was significantly decreased at 60, 120, and 180 min after ventilation in the HI30, HI40, HI50, and HI60 groups (p < 0.05). Compared with the S group, the MAP values were significantly lower at 60, 120, and 180 min after ventilation in the HI30, HI40, and HI50 groups (p < 0.05). Hypercapnia significantly decreased MAP values at 60, 120, and 180 min after ventilation in the HI30 group and at 180 min at the HI40 group (p < 0.05).

**Table 1:** MAP and pH of rats exposed to hypercapnic cerebral hypoxia-ischemia.

Variables	Groups	Baseline	60 min after left CCA ligation	Ventilation with hypoxia/hypercapnia		
				60 min	120 min	180 min
**pH**	S	7.37±0.10	7.38±0.11	7.40±0.05	7.38±0.04	7.37±0.06
	HI30	7.38±0.03	7.35±0.11	7.14±0.07*#	7.11±0.12*#	7.12±0.15*#
	HI30+hypercapnia	7.44±0.03	7.41±0.05	7.10±0.08*#	7.08±0.09*#	7.09±0.08*#
	HI40	7.45±0.11	7.42±0.06	7.11±0.06*#	7.10±0.07*#	7.11±0.04*#
	HI40+hypercapnia	7.39±0.05	7.43±0.07	7.13±0.06*#	7.09±0.05*#	7.08±0.04*#
	HI50	7.38±0.06	7.39±0.04	7.33±0.07	7.28±0.13	7.27±0.11
	HI50+hypercapnia	7.38±0.06	7.41±0.06	7.34±0.07	7.29±0.06	7.27±0.09
	HI60	7.37±0.05	7.40±0.03	7.37±0.05	7.38±0.04	7.36±0.05
	HI60+hypercapnia	7.36±0.11	7.41±0.04	7.35±0.09	7.32±0.10	7.31±0.12
**MAP (mmHg)**	S	119.7±24.0	111.71±14.1	92.0±31.8	84.1±29.1	84.0±35.5
	HI30	129.5±9.1	113.5±17.6	68.8±9.4*#	58.3±8.9*#	56.3±6.8*#
	HI30+hypercapnia	122.5±17.5	111.00±17.2	60.7±4.1*#†	49.0±4.8*#†	47.0±8.4*#†
	HI40	118.7±10.6	101.7±22.4*	75.1±8.6*#	68.7±9.1*#	64.0±7.7*#
	HI40+hypercapnia	115.9±11.9	106.4±18.0	70.7±10.9*#	64.00±9.8*#	55.7±7.8*#†
	HI50	123.6±4.8	100.6±16.6*	85.8±19.5*	84.3±12.9*	76.8±8.4*#
	HI50+hypercapnia	110.7±13.3	110.0±12.6	71.0±14.9*#	74.7±7.6*#	73.7±5.5*#
	HI60	119.00±9.3	119.2±16.7	86.4±10.6*	85.6±8.9*	81.6±9.9*
	HI60+hypercapnia	115.7±27.4	115.0±19.1	92.7±8.0*	85.5±6.7*	84.4±9.2*

Values represent means ± SD of fifteen animals in each group.

S group = rats were exposed to neither hypercapnia nor hypoxia but CCA were ligated.

HI = hypoxia–ischemia. The rats were respectively divided into four subgroups: HI30, HI40, HI50, and HI60 with O_2_ concentrations of 11, 13, 15, or 18% of the gas mixture. HI30 group, exposed to PaO_2_ 30–39 mmHg; HI40 group, exposed to PaO_2_ 40–49 mmHg; HI50 group, exposed to PaO_2_ 50–59 mmHg; and HI60 group, exposed to PaO_2_ 60–69 mmHg. MAP = mean arterial pressure; CCA = common carotid artery

* p<0.05 compared to baseline

# p<0.05 compared to S group

† p<0.05 vs HI group

In addition, at 60, 120, and 180 min after ventilation, the pH values were significantly lower in the HI30 and HI40 groups than in the S group or the baseline values (p < 0.05). Hypercapnia did not significantly alter the pH values at 60, 120, and 180 min after ventilation (p > 0.05).

Compared with the S group, the PaO_2_ values were significantly lower at 60, 120, and 180 min after ventilation in the HI30, HI40, HI50, and HI60 groups (p < 0.05). Hypercapnia treatment did not significantly alter the PaO_2_ values at 60, 120, and 180 min after ventilation in the HI30, HI40, HI50, and HI60 groups (p > 0.05). The PaCO_2_ levels were maintained at 35–45 mmHg in the HI30, HI40, HI50, and HI60 groups, and hypercapnia treatment significantly increased the PaCO_2_ to 60–80 mmHg in the HI+ hypercapnia groups (Table 2).

**Table 2:** PaO_2_ and PaCO_2_ of rats exposed to hypercapnic cerebral hypoxia-ischemia.

Variables	Groups	Baseline	60 min after left CCA ligation	Ventilation with hypoxia/hypercapnia		
				60 min	120 min	180 min
**PaO**_**2**_ **(mmHg)**	S	95.7±5.5	90.7±4.9	85.5±8.8	86.3±6.2	89.4±6.8
	HI30	98.7±8.9	91.8±10.5	38.5±4.8*#	38.5±6.5*#	38.2±9.7*#
	HI30+hypercapnia	98.2±6.1	86.9±5.7	37.5±8.4*#	37.9±5.5*#	34.2±6.2*#
	HI40	96.2±14.5	85.6±11.3	45.8±5.2*#	43.3±4.3*#	44.7±7.5*#
	HI40+hypercapnia	99.3±6.1	86.9±5.1	48.3±3.2*#	45.6±2.2*#	42.7±3.1*#
	HI50	94.3±8.8	86.7±5.7	54.2±3.2*#	55.9±3.4*#	56.1±7.2*#
	HI50+hypercapnia	95.7±11.2	94.0±21.4	56.2±4.4	57.8±6.4	52.7±3.9
	HI60	92.4±18.6	91.6±9.2	66.2±6.1*#	68.9±4.1*#	70.7±4.1*#
	HI60+hypercapnia	95.7±9.7	88.2±8.8	68.7±8.4	70.5±7.8	72.8±3.7
**PaCO**_**2**_**(mmHg)**	S	35.1±7.8	32.8±5.9	37.7±5.8	36.7±7.6	38.4±5.2
	HI30	39.8±12.5	37.7±6.3	40.7±8.9	40.5±8.1	39.9±7.7
	HI30+hypercapnia	36.3±5.9	37.7±2.2	63.6±5.7*#†	70.7±9.8*#†	69.4±4.7*#†
	HI40	37.7±8.9	37.9±6.6	39.6±4.4	38.5±6.7	37.5±6.6
	HI40+hypercapnia	36.8±4.1	34.9±6.6	74.3±6.9*#†	79.3±8.2*#†	72.8±9.2*#†
	HI50	39.5±8.9	38.8±6.7	38.6±5.5	37.1±5.2	38.4±9.3
	HI50+hypercapnia	35.8±7.9	33.7±4.7	65.6±17.4*#†	78.3±13.5*#†	76.8±7.8*#†
	HI60	37.1±5.8	36.6±9.5	36.4±6.6	34.4±8.9	35.2±8.6
	HI60+hypercapnia	38.3±6.9	38.7±4.4	70.6±6.4*#†	72.3±8.9*#†	73.2±5.6*#†

Values represent means ± SD of fifteen animals in each group.

S group = rats were exposed to neither hypercapnia nor hypoxia but CCA were ligated.

HI = hypoxia-ischemia, rats were respectively divided into four subgroups as: HI30, HI40, HI50, and HI60 groups, the O_2_ concentrations were 11, 13, 15, or 18% of the gas mixture to maintain: HI30 group, exposed to PaO_2_ 30–39 mmHg; HI40 group, exposed to PaO_2_ of 40–49 mmHg; HI50 group, exposed to PaO_2_ of 50–59 mmHg; and HI60 group, exposed to PaO_2_ of 60–69 mmHg.

* p<0.05 compared to baseline

# p<0.05 compared to S group

† p<0.05 vs HI group

### Changes in cortex CBF induced by hypoxia and hypercapnia

**Fig 2:**
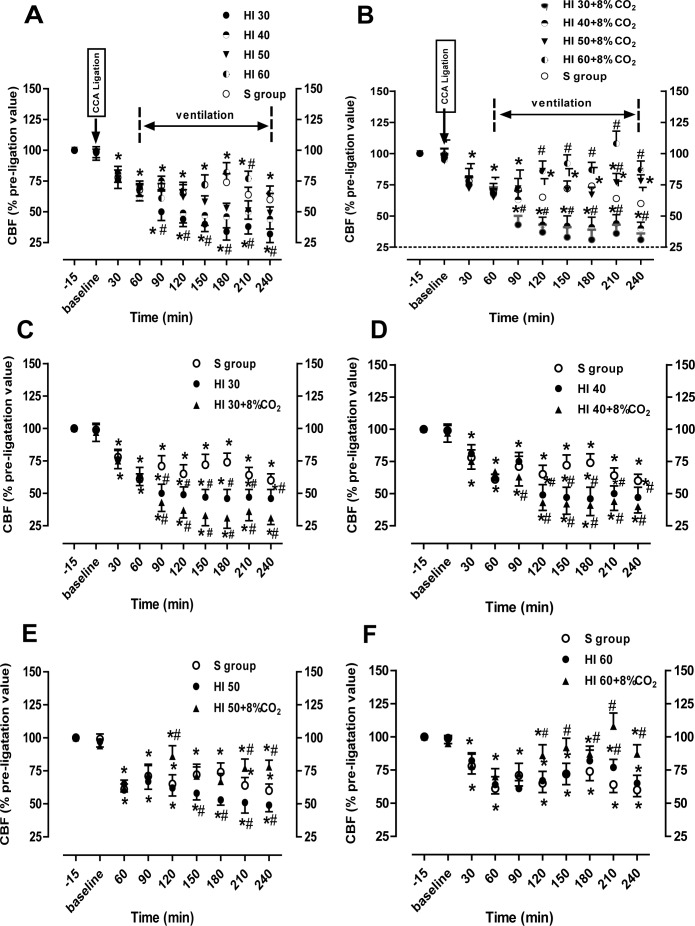
The time-course of changes of cerebral blood flow (CBF). A, B. Changes of CBF in the ipsilateral hemisphere during HI (A) and HI+ hypercapnia (B). C-F. CBF in the S, HI30, and HI30+ hypercapnia groups (C); S, HI40, and HI40+ hypercapnia groups (D); S, HI50, and HI50+ hypercapnia groups (E); and S, HI60, and HI60+ hypercapnia groups (F). CBF was measured before and after common carotid artery ligation. All CBF values are expressed as a percentage of pre-ligation (100%) in each animal. n = 5 for each group. *p < 0.05 compared to baseline; ^#^p < 0.05 vs the S group.

The mean CBF value 10 min before CCA ligation was set as a baseline value. The percentage of the baseline value was used to assess the changes in the cortex CBF at 30–240 min after ligation. Thirty minutes and 1 h after the left CCA ligation, the CBF of the left cortex was decreased by approximately 25–32% compared with the baseline value, respectively (p < 0.05; Fig 2). In the HI or HI+ hypercapnia groups, the CBF values decreased over time in a dose-dependent manner with the greatest decrease in the HI30 group (Fig 2A). In the HI30 and HI40 groups, CBF was significantly decreased by approximately 50% of the baseline values at 1 h after ventilation; after hypercapnia treatment, the CBF values were further decreased by 63% and 57% of the baseline values, respectively (p < 0.05; Fig 2C and 2D). However, after hypercapnia treatment, the CBF values were increased from approximately 50% of the baseline values at 1 h after ventilation to approximately 80–86% of the baseline values at 180 min after ventilation in the HI50 and HI60 (p < 0.05; Fig 2E and 2F).

### Hypercapnia significantly increases BBB permeability and brain water content of rats exposed to severe cerebral HI

**Fig 3:**
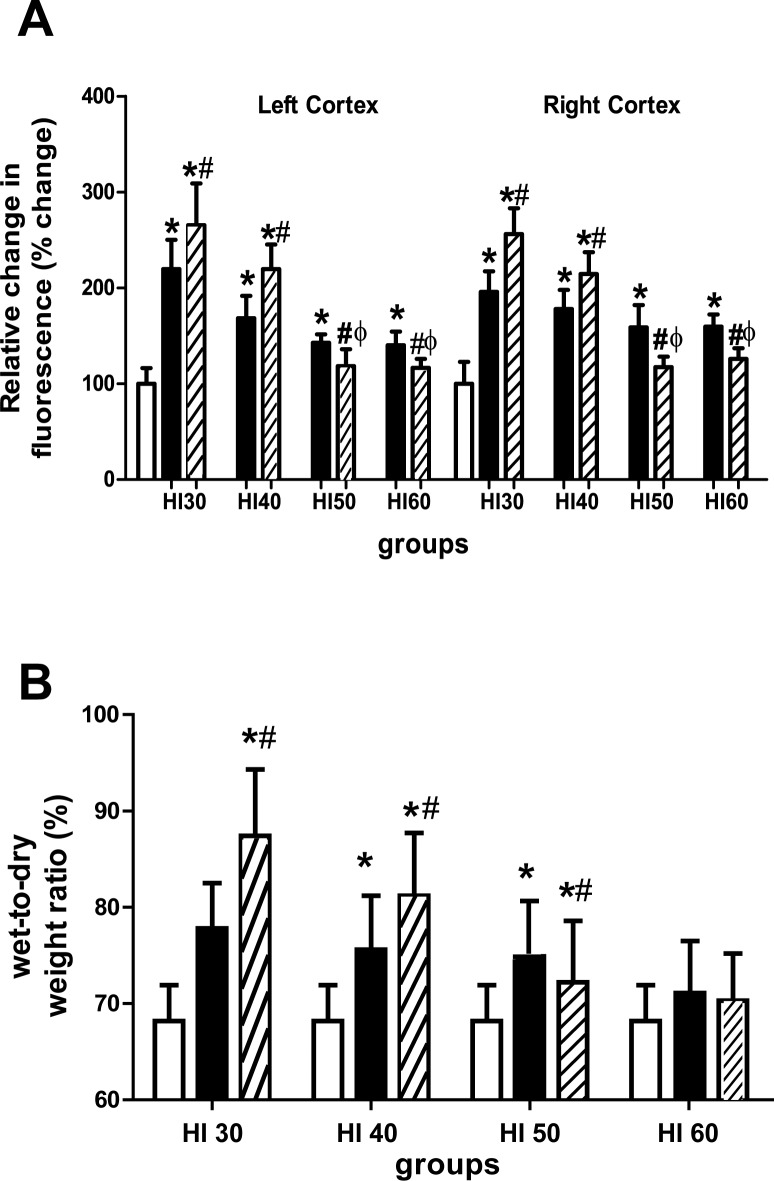
Changes in brain vascular permeability in the cortex and brain edema. (A): The magnitude of blood–brain barrier disruption was quantified by measuring the extent of leakage of 10 kDa dextran. Values are expressed as a percentage of the values in the S group and presented as mean ± SD. (B): Brain water content (wet-to-dry [W/D] weight ratio) in the ipsilateral hemisphere. Clear bars are shams (S group), black bars are hypoxia (HI) and striped bars are hypoxia with hypercapnia (HI+hypercapnia). *p < 0.05 vs the S group; ^#^p < 0.05 vs the HI group (n = 5).

Compared with the S group, the left and right cortex of rats in the HI30, HI40, HI50, and HI60 groups showed increased permeability to 10 kDa fluorescent dextran (p < 0.05; Fig 3A). In the HI30 and HI40 groups, hypercapnia significantly increased BBB permeability (p < 0.05; Fig 3A). In contrary, in the HI50 and HI60 groups, hypercapnia significantly decreased the cortex BBB permeability (p < 0.05; Fig 3A). The brain water contents in the HI30 and HI40 groups were significantly increased compared with the S group, while hypercapnia treatment further increased the water content (p < 0.05; Fig 3B). The brain water content in the HI50 group was significantly increased compared with the S group, while hypercapnia treatment decreased the water content. There was no significantly difference in the water content among the S, HI60, and HI60+ hypercapnia groups (p > 0.05; Fig 3B).

### Hypercapnia treatment increases AQP4 expression in the cortex of rats with severe cerebral HI

**Fig 4:**
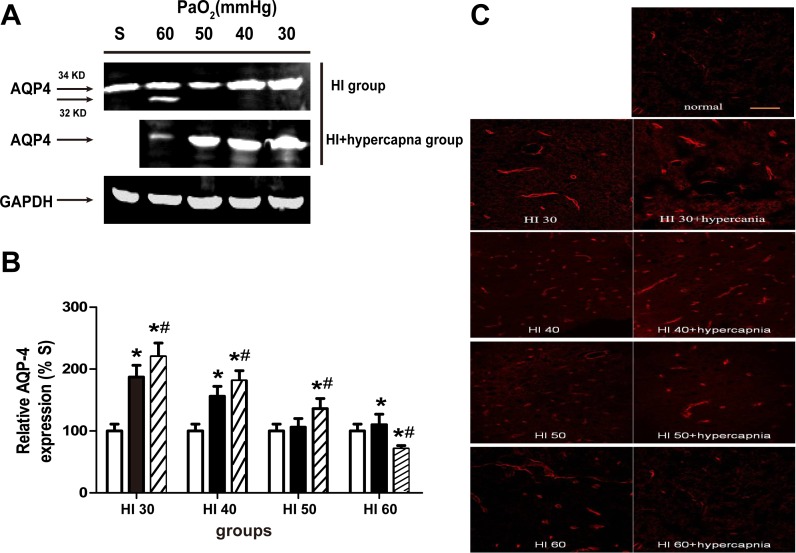
Effects of hypercapnia on the expression of aquaporin-4 (AQP4) in the ischemic cortex of rats in the S group. (A) Western blot analyses of AQP4 expression and (B) densities of AQP4 expression relative to GAPDH in the sham and the HI30, HI40, HI50, and HI60 groups (B) alone or with hypercapnia. Clear bars are shams (S group), black bars are hypoxia (HI) and striped bars are hypoxia with hypercapnia (HI+hypercapnia). Data are expressed as mean ± SD. (n = 3). *p < 0.05 vs. Sham, #p < 0.05 vs. HI groups only. (C) Immunofluorescence labeling of AQP4 in the ischemic cerebral cortex. Magnification, ×20. Scale bar = 100 μm.

Western blot analysis showed that the AQP4 protein levels were significantly higher in the ipsilateral ischemic cortex in the HI30 and HI40 groups than those in the S, HI50, and HI60 groups (p < 0.05; Fig 4A). Hypercapnia treatment significantly increased AQP4 expression in the HI30, HI40, and HI50 groups but significantly reduced AQP4 expression in the HI60 group (p < 0.05; Fig 4B). Consistent with the western blot results, immunofluorescence staining showed that the AQP4 expression in the left cortex was significantly higher in the HI30, HI40, and HI50 groups than in the S group. Hypercapnia increased the AQP4 expression in the HI30, HI40, and HI50 groups but decreased the AQP4 expression in the HI60 group (Fig 4C).

### Hypercapnia treatment attenuated neuronal damage and cell apoptosis of rats with mild to moderate cerebral HI

**Fig 5:**
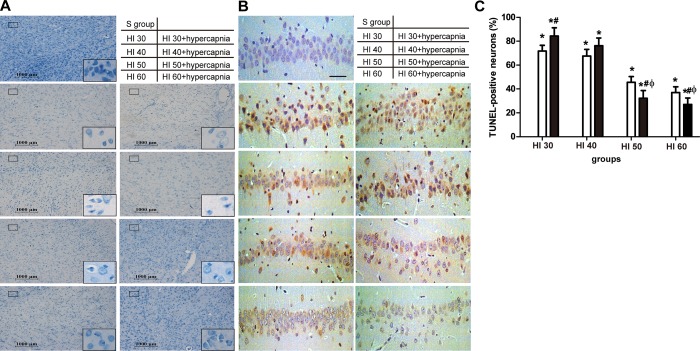
Neuropathological examination. (A) Nissl staining showed more neuronal damage in ipsilateral ischemic cortex of rats in the HI30 and HI40 groups with or without hypercapnia. Hypercapnia treatment afforded neuro-protection against hypoxic-ischemia (PaO_2_ >50) mmHg at 3h after hypoxic-ventilation. Sham animals had no damage. Scale bars = 1000 μm, and = 100 μm in the insets. (B) TUNEL staining revealed that more TUNEL-(+) cells were observed in the HI 30 and HI 40 with hypercapnia groups compared to the HI along groups, but fewer in the HI 50 and HI 60 with hypercapnia groups compared to the HI along groups. Scale bars = 100 μm. (C) Quantification of TUNEL-positive cells in the hippocampal CA1 region. Clear bars are hypoxia (HI), black bars are hypoxia with hypercapnia (HI+hypercapnia) and none bars are shams (S group). *p < 0.05 vs. Sham, #p < 0.05 vs. HI groups only, Фp < 0.05 vs. HI 40+ hypercapnia group (n = 5).

Nissl and TUNEL staining showed that hypercapnia had similar histological findings as in the HI groups (Fig 5). Hypercapnia treatment aggravated neuronal damage in the HI30 and HI40 groups (Fig 5A), but protected cortex neurons from hypoxia-ischemia-induced damage in the HI60 and HI50 groups (p < 0.05, Fig 5A). Also, TUNEL-positive cells in the hippocampal CA1 region of the HI were increased in the HI30, HI40, HI50, and HI60 groups than in the S group (Fig 5B). Hypercapnia treatment significantly increased TUNEL-positive cell counts in the hippocampal CA1 in the HI30 and HI40 groups but reduced them in the HI50 and HI60 groups (p < 0.05; Fig 5C). Neuronal apoptosis in the whole hippocampal area was also detected by Flow Cytometry (FCM) at 3h after hypoxic with or without hypercapnic ventilation (S1 Fig).

## Discussion

We previously found that hypercapnia is a promising therapeutic method for the treatment of cerebral ischemia in rats[14, 15]. In the present study, we further found that the effect of hypercapnia on CBF severity and BBB permeability depended on systemic hypoxia extent. Hypercapnia produced more protective effects against HI-induced brain damage in rats treated with mild to moderate systemic hypoxia (15% and 18% O_2_, PaO_2_ > 50 mmHg) than in rats treated with severe systemic hypoxia of 11–13% O_2,_ PaO_2_ < 50 mmHg). The protective effect of CO_2_ inhalation was associated with the inhibition of hypoxia-induced disruption of cortical CBF and BBB permeability.

Here we used a rat model of hypoxia and ischemia induced by unilateral CCA ligation in combination with systemic hypoxia, which is known to produce brain damage in the hemisphere ipsilateral to the arterial ligation via inducing neuronal death or infarction[24]. Normoxic blood flow in the ligated hemisphere was reportedly unchanged[16], decreased by 10%[25], or decreased by 20%[26]. In the present study, we used a laser Doppler flowmeter to measure rCBF and found that rCBF before and after 30 min and 1 h of ligation decreased to 25 ± 6% and 32 ± 4% of the pre-ligation value, respectively. The long ventilation period (3 h) with normoxic gas resulted in rCBF further decreasing to 40 ± 5% of the pre-ligation value accompanied by a decrease in MAP from 119.7 ± 10.5 mmHg of the pre-ligation value to 84 ± 10.5 mmHg. A decrease in the MAP, in turn, can be associated with a reduced CBF. Systemic hypoxia is reportedly associated with a reduced MAP in rats[27, 28], and an impaired CBF is believed to be the principal pathological mechanism of hypoxia–ischemia[29]. An increased MAP can lead to an increased CBF, thus alleviating the degree of neurological dysfunction during acute ischemic stroke[30]. Here we found that the MAP and CBF decreased to a greater degree in the ligated hemisphere rats treated with severe hypoxia (PaO_2_ < 50 mmHg) than in those treated with mild to moderate hypoxia (PaO_2_ > 50 mmHg), suggesting that hypoxemia applied after unilateral CCA ligation reduced the systemic blood pressure and hypotension along with hypoxemia likely contributed to a greater decline in CBF. This may be due to impaired autoregulation and reduced cerebrovascular reserve under severe hypoxia[31, 32]. In the present study, we found that hypercapnia treatment resulted in CBF recovery in rats treated with mild to moderate hypoxia (PaO_2_ > 50 mmHg) but not in rats treated with severe hypoxia (PaO_2_ < 50 mmHg) accompanied by a MAP decrease. Presumably, severe hypoxia produces more severe cardiovascular depression than mild hypoxia, thus reducing hypercapnia-induced CBF recovery. Carbon dioxide is a very potent modulator of CBF, and hypercapnia causes the cerebral vessels to vasodilate [33].Ken’s study indicate that the hypercapnic vasodilatation depends on cyclooxygenase-1 (COX-1)-derived prostaglandin E2 (PGE2) acting on EP1 receptors and highlight the critical role that COX-1-derived PGE2 and EP1 receptors play in the hypercapnic regulation of the cerebral circulation[34].

Hypoxia–ischemia leads to necrotic cell death, inflammation, brain edema, excitotoxicity, and apoptotic cell death [35, 36]. Because the pyramidal neurons in the hippocampal CA1 region are vulnerable to cerebral ischemia[37], so we assess the effect on apoptosis in our study, the hypercapnia therapy has also been reported to have an antiapoptotic effect by our previous work [14]. The results of the present study suggested that hypercapnia significantly promotes the intact hippocampal neuronal apoptosis in rats with severe hypoxemia but inhibited cell apoptosis in rats treated with mild to moderate hypoxia (PaO_2_ > 50 mmHg; S1 Fig). BBB disruption permits the extravasations of albumin and other high-molecular-weight compounds, resulting in edema formation and increased intracranial pressure (ICP)[38]. Willis et al. reported that hypoxia rapidly increased hippocampal vascular permeability to dextrans and rat immunoglobulin G [39]. This study’s findings highlight the great differences in vulnerability of cortical and hippocampal vascular integrity in response to different degrees of hypoxia (180 min), which may reflect differences in BBB permeability in the two brain regions in response to different degrees of hypoxia. AQP4 may have an important role in aiding the passage of water through the BBB, and therefore upregulation of AQP4 may be a molecular mechanism underlying brain edema[40]. It has previously been demonstrated that AQP4, which is located on both sides of the BBB, is associate with reactive gliosis[41]. Lin et al reported that the protein expression levels of AQP4 in the low levels of O_2_ (9–11%) and high levels of CO_2_ (5–6%) conditions was significantly increased[42]. Although we did not find evidence that AQP4 expression is associated with reactive gliosis at such an early stage into lesion development, severe hypoxia with hypercapnia resulted in a higher brain water content and AQP4 expression level than mild or moderate hypoxia can be confirmed in the present study. However further experiments are required to investigate this.

In the clinic, hypoxemia is defined as a PaO_2_ < 60–65 mmHg and is reportedly associated with worse outcomes after severe traumatic brain injury (TBI) [1, 43]. In animal models of TBI, a PaO_2_ of 40 mmHg[44] but not 60 mmHg[45] increases neuropathology. Although we used different animal models of brain injury, we found that mild hypoxemia produced minimal neuropathological damage, whereas severe hypoxemia increased both brain edema and death in selectively vulnerable neurons. It is possible that the severe injury exceeded the therapeutic potential of short-term exposure to hypercapnia. The large volume of ischemic tissue may have overwhelmed any possible benefit of hypercapnic treatment. Therapeutic hypercapnia has been proven to play a potential neuroprotective role in rat models of middle cerebral artery occlusion–reperfusion [15, 46] and global cerebral ischemia [14]. However, we previously found that the extent of respiratory acidosis is dose-dependent on the PaCO_2_ level (e.g., after 30 min of mild, moderate, and severe hypercapnia, the arterial pH was 7.21 ± 0.07, 7.13 ± 0.09, and 7.05 ± 0.1, respectively) [14]. Therefore, the effect of hypercapnia on brain damage may be dependent on the degrees of systemic hypoxemia and cerebral pH. Severe hypoxia and low pH may contribute to the lesser neuroprotective effect of hypercapnia.

This study has some limitations. First, ICP was not measured in this study since we found that its measurement may increase the mortality rate and the complications associated with the CBF measurement after HI. In fact, our previous study demonstrated no statistically significant differences in ICP among different hypercapnia levels used in this study at 3 h after reperfusion onset, although the neuroprotective effects were observed only in rats treated with mild and moderate hypercapnia in a transient global cerebral ischemia–reperfusion model[1414]. Second, we reported histologic outcome variables for 4 h after hypoxia ischemia, which may not reflect long-term outcome. Nevertheless, our results suggest that the addition of hypercapnia contributes to worse injury after CCA ligated along with severe hypoxemia (PaO_2_ < 50 mmHg).

## Conclusion

In conclusion, here we found for the first time that the potential beneficial and detrimental effects of CO_2_ may be largely dependent on systemic hypoxia exposure extent. The exposure of HI rats to severe hypoxia resulted in worse ischemic brain injuries than those resulting from mild or moderate hypoxia during the same level of hypercapnia (PaCO_2_ of 60–80 mmHg). Our finding that the maintenance of sub-normal PaO_2_ with mild to moderate hypercapnia during operative ventilation may be beneficial for improving CBF and BBB function and thus alleviate HI-induced brain injury.

## Supporting Information

S1 Fig Neuronal apoptosis in the hippocampal area was detected by Flow Cytometry (FCM) at 3h after hypoxic with or without hypercapnic ventilation. The level of apoptotic neurons was measured by flow cytometry (1, 2, 3, and 4 quadrants represent dead neurons, late apoptotic neurons, normal neurons, early apoptotic neurons, respectively). A: The results of apoptotic neurons in sham, HI, and HI+Hypercapnia groups, respectively. B: The percentage of apoptotic neurons of different groups at 3h after hypoxic ischemia. The distribution of apoptotic neurons showed that few apoptotic neurons were found in the Sham group, more apoptotic neurons were induced in the HI groups, and the level of apoptosis was significantly decreased in the HI 50 and HI 60 with hypercapnia groups compared to the HI along groups, but increased in the HI 30 and HI 40 with hypercapnia groups compared to the HI along groups. *p < 0.05 vs. Sham, # p < 0.05 vs. HI groups only, Ф p < 0.05 vs. HI 40+ hypercapnia group (n = 4).
(TIF)

S2 Fig HE Staining. A. Representative hematoxylin and eosin staining showing partial cell death, neuronal loss, cell shrinkage, nuclear condensation, and fragmentation. Magnification, ×400. Scale bars = 100 μm. (B) The percentage of surviving neurons. The percentage of surviving neurons in the ischemic ipsilateral cortex was significantly decreased in the HI30, HI40, HI50, and HI60 groups compared with the S group (p < 0.01). Hypercapnia treatment aggravated neuronal damage in the HI30 and HI40 groups (p < 0.05), but protected cortex neurons from hypoxia-ischemia-induced damage in the HI60 and HI50 groups (p < 0.05). *p < 0.05 vs. Sham, #p < 0.05 vs. HI groups only, Фp < 0.05 vs. HI 40+ hypercapnia group (n = 5).
(TIF)
