# Supplementary figures and images for: Effects of Acute Systemic Hypoxia and Hypercapnia on Brain Damage in a Rat Model of Hypoxia-Ischemia
Source: PLoS One. 2016 Dec 1;11(12):e0167359. doi: 10.1371/journal.pone.0167359 (PMC5131999; doi:10.1371/journal.pone.0167359)

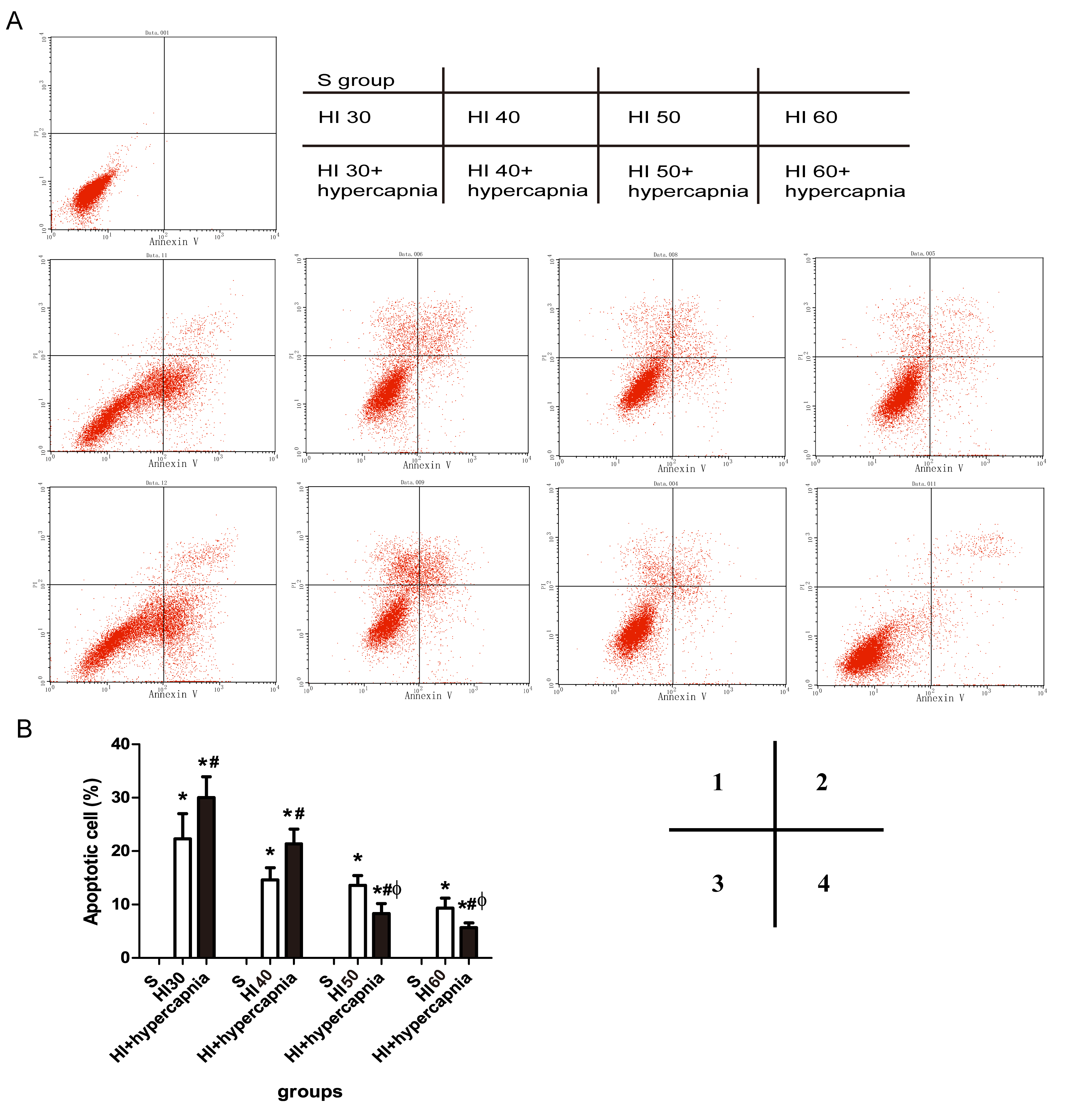

Supplement: S1 Fig — The level of apoptotic neurons was measured by flow cytometry (1, 2, 3, and 4 quadrants represent dead neurons, late apoptotic neurons, normal neurons, early apoptotic neurons, respectively). A: The results of apoptotic neurons in sham, HI, and HI+Hypercapnia groups, respectively. B: The percentage of apoptotic neurons of different groups at 3h after hypoxic ischemia. The distribution of apoptotic neurons showed that few apoptotic neurons were found in the Sham group, more apoptotic neurons were induced in the HI groups, and the level of apoptosis was significantly decreased in the HI 50 and HI 60 with hypercapnia groups compared to the HI along groups, but increased in the HI 30 and HI 40 with hypercapnia groups compared to the HI along groups. *p < 0.05 vs. Sham, # p < 0.05 vs. HI groups only, Ф p < 0.05 vs. HI 40+ hypercapnia group (n = 4). (TIF) [file pone.0167359.s001.tif]

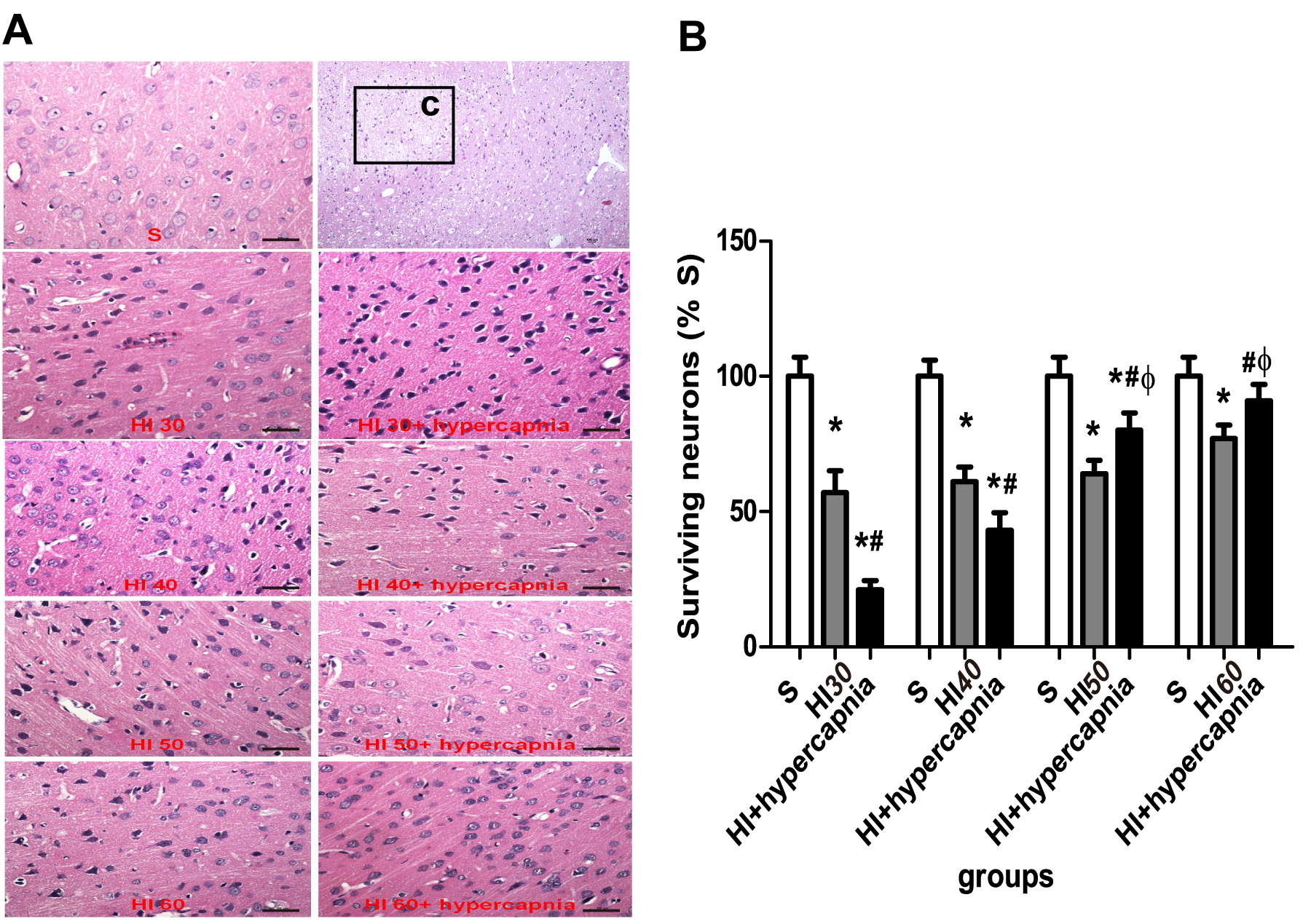

Supplement: S2 Fig — A. Representative hematoxylin and eosin staining showing partial cell death, neuronal loss, cell shrinkage, nuclear condensation, and fragmentation. Magnification, ×400. Scale bars = 100 μm. (B) The percentage of surviving neurons. The percentage of surviving neurons in the ischemic ipsilateral cortex was significantly decreased in the HI30, HI40, HI50, and HI60 groups compared with the S group (p < 0.01). Hypercapnia treatment aggravated neuronal damage in the HI30 and HI40 groups (p < 0.05), but protected cortex neurons from hypoxia-ischemia-induced damage in the HI60 and HI50 groups (p < 0.05). *p < 0.05 vs. Sham, #p < 0.05 vs. HI groups only, Фp < 0.05 vs. HI 40+ hypercapnia group (n = 5). (TIF) [file pone.0167359.s002.tif]
